# Mutual Role of Patients and the Healthcare System in the Control of Cutaneous Leishmaniasis

**DOI:** 10.1155/2023/7814940

**Published:** 2023-08-26

**Authors:** Mehdi Bamorovat, Iraj Sharifi, Setareh Agha Kuchak Afshari, Pooya Ghasemi Nejad Almani

**Affiliations:** ^1^Leishmaniasis Research Center, Kerman University of Medical Sciences, Kerman, Iran; ^2^Medical Mycology and Bacteriology Research Center, Kerman University of Medical Sciences, Kerman, Iran

## Abstract

Leishmaniasis is a neglected and old emerging/reemerging disease that has extremely increased and expanded in the different geographical areas. Cutaneous leishmaniasis (CL) is a common global public health concern in the tropics and subtropics reported from the Eastern Mediterranean Region, Africa, Latin America, and the Indian subcontinent. The presence of numerous epidemiological and clinical characteristics of the disease present a challenge in managing and controlling it. Despite wonderful efforts to control the disease in endemic areas, it appears that the burden of CL is still high. There is no comprehensive survey considering the interactive role of patients and medical services in the international CL control system. In the present study, the effective and mutual responsibility of the patients and the healthcare management in controlling the CL are reviewed, described, and discussed. Some patient-related factors that have a remarkable impact on the proper treatment and control strategies are low-socioeconomic condition, lack of sufficient knowledge about the disease, absence of personal protection, disregarding adherence to treatment, culture and incorrect beliefs about disease and therapy, depression, and lack of appropriate support from the family. On the other hand, some healthcare system-related factors that have an important effect on the suitable therapy and management of the disease are the economic burden of healthcare and drug preparation, unsuitable follow-up assessments, health education, early detection, and the right treatment, handling of unresponsive cases, active case detection, and monitoring/evaluation. Improving the socioeconomic conditions and living standards supports longstanding elimination approaches, and facilitates the wide-ranging control of the disease. Trained health staff and experienced clinicians should strengthen the building capacity for appropriate control programs throughout the healthcare system. These measures need sustained encouragement and appropriate multifunctional activities through all government segments including policymakers, the health system, clinical physicians, researchers, and also the private sector. In a suitable strategy, it is essential to understand precisely all the aspects of control strategies to respond correctly to the requests. Nevertheless, due to the complexity of the causative agent, major challenges/gaps remain, and vigorous conservative control plans should be continued until novel tools become available.

## 1. Introduction

### 1.1. Epidemiology and Control

Leishmaniasis is one of the major neglected and tropical infectious diseases. It has extensively increased, silently expanded in various geographical areas, and spread among diverse hosts and vectors [1, 2]. Almost 23 *Leishmania* species have been reported for the human infection [3]. The disease is transmitted by an infected female sandfly bite to reservoirs (humans, rodents, and/or canines). People are infected incidentally when they go into endemic regions. The disease is endemic in various areas in more than 100 countries [4].

Currently, more than 1 billion inhabitants have been at risk of infection worldwide [4]. Cutaneous leishmaniasis (CL) is an important public health concern in the endemic countries and territories. Various epidemiological characteristics and clinical forms demonstrate a challenge in managing the disease [5]. Although the visceral form (visceral leishmaniasis; VL) is a fatal type of disease, CL is the most common form universally [6]. Eighty-nine countries are affected by the endemic CL in the Eastern Mediterranean Region, Africa, Latin America, and the Indian subcontinent [7]. In general, the annual new cases are assessed to be about 0.7–1 million. In 2019, the Global Burden of Disease study expected the prevalence of CL to be 4.6 million cases [7, 8]. In 2020, from seven countries: Algeria, Brazil, Colombia, Afghanistan, Pakistan, Iraq, and the Syrian Arab Republic, nearly 80% of CL have been reported [7]. Overall, over 82% of the disease caseload belong to the Eastern Mediterranean Region [7].

**Figure 1:**
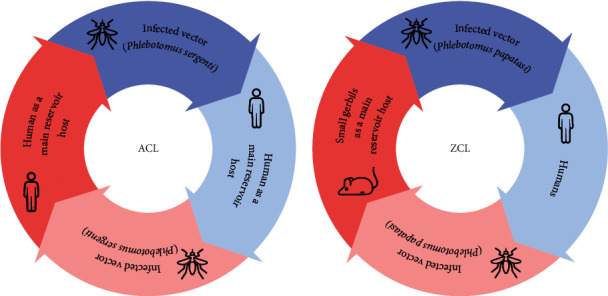
The comparison of life cycles and host transmission of anthroponotic cutaneous leishmaniasis (ACL) and zoonotic cutaneous leishmaniasis (ZCL).

In the Old World, *Leishmania major* is responsible for the zoonotic CL (ZCL) form where rodents are the main reservoir host and *Phlebotomus papatasi* is the primary sandfly vector. Humans play an incidental role in the epidemiology of this species [9]. While *L. tropica* is accountable for the anthroponotic CL (ACL), *Ph. sergenti* is an important vector, and humans are the chief source of infection (Figure 1) [9]; although, dogs might be implicated in the life cycle of the parasite [10].

Systematic collection and analysis of data related to CL occurrence in inhabitants are essential for planning, implementation, monitoring/evaluation, and reporting in routine public health practice. Among many things, surveillance data are vital to determine disease burden and trends over time and in endemic countries; to monitor disease extension into nonendemic areas; to identify the boundaries of autochthonous transmission within each endemic country; to detect epidemic clusters; and to monitor and evaluate efforts toward appropriate case management and control strategies. The government's responsibilities in the control of leishmaniasis include improving the quality of life of people and desertification, fighting against animal reservoirs, improving the environment, and providing drugs with fewer side effects and parallel treatments [11].

Despite great efforts over decades to control CL in the endemic countries, it appears that the CL rate is still high. This statement shows that the basic infrastructures and the past support and present conducted plans associated with diagnostics, treatment, and care are inadequate [12]. In endemic foci, the intricacy of the zoonotic and anthroponotic life cycles, numerous species of sandfly vectors, several reservoir hosts, diverse species of the parasite, environmental and demographical factors, variable responses to treatment, and various clinical forms are the main challenges/gaps in the epidemiological and control fields of CL [13–17]. In this respect, the behavior of patients and the healthcare system as two basic pillars for disease control are of great importance. Nonobservance of health-treatment principles by patients and the healthcare surveillance system and other aggravating factors lead to the creation of suitable conditions for the propagation of the disease cycle and its lack of control in the region. CL control is a multidimensional challenge of various compound issues. The neglected topics in this challenge are the interaction of patient-related factors and healthcare system-associated factors.

The objective of this study was to review the effectiveness and mutual role of the healthcare system and the patient-related factors in the control strategies of CL, particularly ACL.

### 1.2. Leishmaniasis as a Neglected Tropical Disease

Neglected tropical diseases (NTDs), such as leishmaniasis, are known as “neglected,” since they commonly affect the world's poor and generally have not received as much consideration as other diseases. NTDs tend to expand in developing areas of the world, where water quality, sanitation, and access to health care are insufficient and there are political problems and other major challenges [18–21]. Though, some of these diseases are also observed in regions of the United States with high degrees of poverty [22]. NTDs suffer a remarkable fee on the global health. The World Health Organization (WHO) assesses that more than 1 billion people get from at least one NTD [20]. Though NTDs seldom lead to death, they can cause considerable disability that continues for a lifetime, including blindness, fatigue, and disfigurement. NTD cases miss school, are incapable to work, or are too turbulent to seek medical care. By reducing the quality of life and prospects of success, NTDs can strengthen the cycle of poverty among the world's poor people [18, 23].

### 1.3. Cutaneous Leishmaniasis Skin Lesions

**Figure 2:**
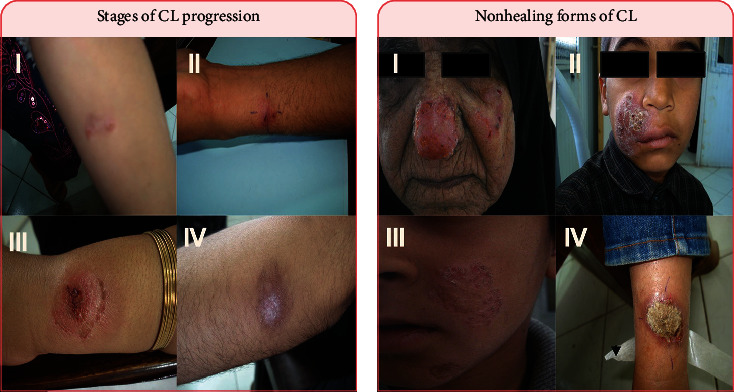
Patients with CL lesions display various clinical features of healing and nonhealing skin lesions. (a) Illustrates the progress of CL lesion stages: I. papule, II. nodule, III. ulcer, and IV. scar; and (b) presenting the nonhealing forms of CL (I–IV).

The clinical appearances of different forms of CL have been categorized in many ways. The majority of authors have conventionally divided ZCL and ACL into two separate types of “wet” or rural type and “dry” or urban type, respectively, regardless of pathogenesis [24]. CL evolves in four main clinical features. Acute or localized CL signifies the most characteristic clinical presentation of the illness worldwide. The lesion starts as an erythematous papule after 1–2 months, then develops into a painless and much larger nodule that involves the deeper skin layers. The nodule may progressively ulcerate over a period of 2 weeks to 6 months to a volcanic form. Spontaneous healing finally results in a depressed cutaneous scar, distress, life-long stigmatization, permanent skin alterations, and enduring protection from the disease (Figure 2(a)). Acute CL may evolve into one of the several clinical forms of the disease including nonhealing forms depending on the complexity of the host's immune status, causative agent, and varied biological interactions between the parasite and the main reservoir host (Figure 2(b)) [25–27].

Clinical assessment of CL lesions along with the accurate identification of its leishmanial species is critical for selecting suitable treatment schedules and strategy preparation, notably in ACL areas wherein interventions are restricted to primary detection and effective therapy of cases [28]. Since CL mimics other circumstances, predominantly infectious and noninfectious conditions, knowledge of the disease is vital for dermatologic practitioners in the affected areas [28].

### 1.4. Antileishmanial Drugs

Meglumine antimoniate (MA, Glucantime®) is the gold standard medication for ZCL and ACL, used intralesionally (every week for 8–12 weeks in combination with biweekly cryotherapy) and intramuscular route (every day for 21–28 days) [29, 30]. According to WHO guidelines, usually, ZCL patients should not be treated except if vital body parts are involved. But ACL cases need to be treated due to this form being anthroponotic and the only reservoir host is human [3, 31]. The treatment modality is assumed by several standards such as the size, number, and location of the lesion, species of *Leishmania*, and the immunologic condition of the patient. In a time of restriction and contraindication for choice drug therapy, other drugs such as lipid formulations of amphotericin B (AmBisome®), paromomycin, miltefosine, ketoconazole, itraconazole, and fluconazole and also multiple therapies as a combination, could be used [31]. In general efficacy of MA against ACL, is high; however, cases of treatment failures (unresponsiveness) have been reported [25, 32–35].

## 2. Study Overview and Prospect

This review mostly focuses on the studies available from 1995 to 2023 on the CL control determinants, particularly on patients and the healthcare system as two key important factors worldwide. A comprehensive literature search was conducted using the database of PubMed, Google Scholar, and MEDLINE. Different inclusion and exclusion criteria including date, type, and quality of publications, language, exposure interest, and reported outcome were used for particular concepts in the research sources. The obtained sources were evaluated and reviewed by all the authors based on the research criteria and questions, and finally, the desired results were extracted.

For this aim, a literature review, research articles, and books were searched by the subsequent keywords: “control,” “cutaneous leishmaniasis,” “cutaneous leishmaniasis control,” “leishmaniasis,” “anthroponotic cutaneous leishmaniasis,” “CL patients,” “zoonotic cutaneous leishmaniasis,” “treatment,” “healthcare system,” “health system,” “treatment failure,” “*Leishmania tropica*,” “*Leishmania major*,” and “meglumine antimoniate”. It is worth noting that CL is an endemic and notifiable disease throughout the world. However, there is no comprehensive survey into the mutual action of CL patients and the healthcare services in the CL control worldwide. In this study, the practical and interactive role of patients and the healthcare system in controlling CL were reviewed, described, and discussed.

## 3. Patient-Related and Healthcare System-Related Factors

### 3.1. Patient-Related Factors

#### 3.1.1. Poverty and Socioeconomic Determinants

Commonly, one of the main factors that are related to the high severity of infectious diseases is poverty [36]. Leishmaniasis as a major neglected parasite disease has a related potency to poverty and together they make a harmful cycle [36, 37]. Leishmaniasis frequently affects people living in the needy parts of developing countries and places extra economic stress on poor conditions for families [38–40]. Poor housing conditions and low-living standards result in peridomestic areas and increase the risk of transmission. This condition provides a suitable place for the growth and reproduction of sandflies. People such as laborers who live in poor households and low income, are at risk of transmission [3]. Also, they sleep in a shared room and attract anthropophilic sandflies; hence, poverty rises the risk of CL. Hygiene circumstances of such housing, including the loss of sewage treatment plants and solid waste management, could supply resting places for vectors, raise the potential of sandflies' reproduction, and also comfort contact with the humans [41]. Vectors (sandflies) are mostly possessed in the crowded houses, which prepare a suitable source of blood meals. Some other socioeconomic factors of patients that have a remarkable effect on the treatment outcomes are unstable conditions of living, unemployment, and the high cost of drugs [42, 43].

A long distance from the treatment center (living in the suburbs of the cities) and the high cost of transportation are other socioeconomic factors that are related to poverty. In a study in Peru, the authors showed a significant relationship between poverty and the occurrence of CL cases [43]. Mohamed El Alem et al. [44] confirmed the significance of socioeconomic factors, especially poverty on the distribution and incidence of CL. In southeastern Iran, Bamorovat et al. [16] and Bamorovat et al. [40] displayed that the poor interior housing condition as a socioeconomic factor can be affected by creating unresponsive CL cases due to *L. tropica*.

Also, illiteracy and low level of education have been demonstrated to impact the incidence rate of the disease [45]. Generally, a high level of education raises awareness of diseases and expands the control procedures. Certainly, it will be more convenient and more effective to educate the literate and with a higher level of education to better understand the aspects of disease control. A study in Palestine displayed that lower incidences of CL were associated with a high level of education and knowledge of the household head [46]. According to the above explanations, the role of socioeconomic conditions, especially poverty, is very effective in the prevalence and incidence of CL and, as a result, in controlling the disease. Therefore, the role of governments and health officials in improving economic and health conditions, respectively, is undeniable. It is the government and executive authorities who provide and manage the budget and necessary facilities to improve the health status of society.

#### 3.1.2. Family Support

The family is the main and important social organization for health progress. People are born and take resources for their growth and improvement. It has an early influence on the health and development of individuals. The family influences healthy manners and assists in the improvement of chronic diseases [47]. It is generally linked to positive health subsequences. Families are especially shown to be more preventive for the psychological health of children. They provide the support and facilities needed for healthy living, prevention of disease, and preparation conditions for early detection and treatment. It is the base for the healthy and mental health development of the individuals. Therefore, the lack of proper support from the family can be a risk factor affecting the public health and spread of disease in society [47].

Those families whom themselves have sufficient mental and physical health and social welfare can support their patients for recovery and healing. The existence of an ideal and healthy family that supports its members depends on an ideal and well-being society and government that provides for economic and psychological needs. Peñarrieta et al. [48] showed that family support has a significant role in reinforcing the management of diseases in the persons with chronic diseases. Studies performed in China and Indonesia showed that during the COVID-19 pandemic, family support has an important and positive role in preserving the mental health and recovery from the disease [49, 50]. Also, investigations showed the significant role of family support in promoting health and reducing the risk of illness, preventing disease onset, influencing health care and treatment decisions, helping members highly susceptible to stress, and care and recovery [51, 52]

#### 3.1.3. Personal Protection Measures

People can protect themselves with repellents including insecticide–impregnated nets (pyrethroids), diethyltoluamide (DEET), fabrics, clothing, and windows and door net. Though, their helpful role has not been well studied in the different areas [53]. Insecticide bed nets have been demonstrated to be impacted the control of the disease but on the other hand, many families living in endemic areas due to low income cannot provide bed nets [54, 55]. In Afghanistan, approximately, 78% of undertakers stated that they could not provide bed nets [36]. The behavior of sleeping outside could also raise the risk of sandfly bites. Such risk-related factors are mostly considered the reason for epidemics [44, 56, 57]. Applied insecticides to decrease the population of sandflies are efficient in reducing the new cases of CL [58]. However, there is insufficient documentation to inform which of them is better than the other, personal protection using insecticide-treated bed nets, sheets, or curtains or applying insecticides to spray the interior walls of houses [58].

Long-lasting insecticide nets have been displayed to be effective for CL in many regions sandflies of the world [59]. A study performed in Bangladesh showed a 66.5% decrease in disease incidence after the impregnation of available bed nets with insecticide [60].

Overall, vector control assistances to decrease or interrupt the transfer of leishmaniasis by reducing the population of sandflies. As mentioned above, this approach consists of using insecticide, protection by net, environmental management, and particularly personal protection [61].

#### 3.1.4. Adherence to the Treatment

There is a strong reason that many cases with chronic diseases, such as diabetes, hypertension, asthma, immunodeficiency, and organ problems, and on the other hand, drug interactions and side effects have suffered following their treatment regimens and sometimes do not adhere to treatment. One of the major reasons for treatment failure despite clinical care is low adherence to treatment [62–64]. This fact affects the psychological problems, decreases the patient's standards of living, and is a misuse of healthcare system efforts. The outcome of weak adherence is long treatments, unpleasant health results, and increased healthcare costs [64, 65]. Adherence is one of the main elements of the effectiveness of therapy because nonadherence to it decreases optimal medical benefits [62, 64, 66]. In developed countries, adherence to therapy for chronic diseases is estimated to be 50% [47]. In Colombia, a study conducted on infected adults and children with CL (considering all treatment regimens) showed that the nonadherence rate was 9.5%. that associated with raised odds of nonhealed CL cases (OR: 3.59) [67]. Rodrigues et al. [68] displayed that poor adherence is a key independent factor for treatment failure, in this regard, nonuniform therapy was a determining factor in the CL consequence (RR: 1.85). In Nepal, ideal obedience to miltefosine treatment for VL was reported in 57.9% of cases. Treatment adherence was better in patients that were knowledgeable about drawbacks compared with cases that were not [69]. Bamorovat et al. [64] showed that the most considerable feature that was important in poor treatment adherence was complex treatment regimens. Patients who had chronic diseases and after that are infected with leishmaniasis must entail a new therapy administration on the timetable of their preceding treatment regimen. There is strong evidence that many cases with underlying diseases have difficulty following their new schedules [64].

Generally, this problem weakens the ability of healthcare staff worldwide to achieve population health aims. Treatment adherence is a multidimensional therapy problem that is created by the interaction of several related factors between patients and healthcare personnel. Since ACL cases need to be treated due to their anthroponotic form and the only reservoir host is human, it seems adherence to regular treatment on behalf of all patients is essential for controlling the disease.

#### 3.1.5. Modifying Cultural Beliefs

Some CL cases fail their therapy because of the traditional treatments and other drugs, and the main therapy is abandoned due to the incorrect beliefs and culture surrounding the disease and therapy. These cases frequently abandon the standard treatment (MA) due to the pain and distress of the injection and remain using traditional remedies [47]. Differences in cultures, religions, and beliefs throughout the world make it challenging for healthcare systems to provide medical care. Beliefs around drugs and knowledge of chemical treatment and diseases differ among various cultures [70].

In Suriname, results of an investigation showed that cases with CL, use potentially damaged nonbiomedical materials including dilute sulfuric acid, gasoline, insecticides, and herbicides to treat their wounds [71]. On the other hand, the incorrect belief of CL in Afghanistan that believe the disease can be physically transmitted through human-to-human leads to social exclusion [72].

Improving correct information of patients about their illness and their treatment can positively influence their therapeutic outcomes. Practical strategies of health authorities to solve these problems include providing cultural training and developing therapeutic policies that reduce barriers to providing patient care [73–75].

#### 3.1.6. Reducing Mental Disorders

Lesions of CL can be a reason for mental distress, such apparent lesions have been correlated with the social stigma that possibly leads to loneliness and self-stigma, and psychosocial morbidity [76–78]. Psychological distress has been shown to interrupt treatment adherence. Anxiety, depression, and how people manage their stress are the main important determinants of treatment obedience [79–81]. These undesirable approaches can reduce the stimulus to contemplate for her/his self and hurt the ability of cases to follow treatment [80, 82]. Leishmaniasis also harmfully impacts the mental and social situation of females. The atypical lesion leads to social stigmatization, disconnect with the community association, unable to marry, and may lead to psychological disorders [83, 84]. On the other hand, married women hurt rejection from their husbands/partners who are frightened of having the infection from them [85]. Moreover, CL lesions impact a woman's ability to take maintenance of her kids and do daily household responsibilities and finally have a significant impact on their psychological health [85].

In a study conducted in Turkey, cases with CL had significantly higher levels of depression and anxiety and were less pleased with their appearances compared to the control group [86]. Social distress, anxiety, and depression can be some of the main recognizable demonstrations of neglected diseases specifically in CL cases [87]. Also, depression and other psychological disorders can activate inflammation, consequently motivating more cytokines to raise immune response to the pathogen as well as stressor factors [88]. Together stress and depression raise the risk of prolonging infections, increasing infectious agents, and therefore raising the creation of treatment failure forms [89, 90]. Therefore, management of these critical problems is vital to treatment outcomes as well as control.

#### 3.1.7. Promoting Public Awareness about the Diseases

Some of the patient-associated issues in proper CL therapy for control include the knowledge and understanding, behaviors, and type of patient view [91]. Patients' awareness and opinion toward their disease, self-efficacy to be involved in the management of the disease, positive views regarding the treatment outcomes, and knowledge of the consequences of poor adherence are some significant factors to proper treatment as well as control [92, 93]. CL characteristically clusters in marginalized societies in focal neighborhoods. It is imperative to upsurge the awareness of the disregarded public about the disease control strategies. Exact, adapted communications of health education should be developed. Community involvement is critical to maximizing the impact of control approaches, including case detection, control of principle vector control, and main reservoir hosts. A good exchange of ideas between community leaders and the public must be established [93].

Studies in Yemen and Saudi Arabia had been shown that a poor rate of knowledge about the CL and its epidemiological aspects existed among rural endemic societies. This problem together with the inefficient healthcare systems may be contributing to the continued CL endemicity [94, 95]. The results of a study in southern Iran showed that incomplete knowledge of the society about various epidemiological aspects of CL needs health education to enhance the awareness of the community about CL [96]. Also, an evaluation of the CL-endemic region of Iran showed that the total awareness score of participants from the disease was 17.47 out of a possible 30 [92].

People's knowledge and awareness of leishmaniasis have an influence on prevention and control in the endemic regions [96]. Dires et al. [97] in Northeast Ethiopia showed that almost three-fourths of participants had poor awareness regarding CL, and two-thirds of them had an unsuitable attitude to the disease. On the other hand, in an investigation, the results were shown that in CL cases, the awareness of CL signs, vectors, and reservoirs was more than the awareness of preventive and control procedures [98]. Also, similarly to CL, the findings of another disease (Diabetes mellitus) had shown increasing knowledge of patients about their disease can positively affect their treatment adherence and its outcome [99]. Hence, it is recommended that health education on CL transmission, prevention, and control should be prepared in the endemic areas.

### 3.2. Healthcare System-Related Factors

#### 3.2.1. Supporting Healthcare Economic Burdens

Many challenges/gaps in the healthcare system potentially affect program control strategies. These factors are included the high-economic burden, logistic difficulties in achieving initial healthcare centers, and treatment costs. The treatment and cost of providing drugs for leishmaniasis are very expensive compared to other diseases [100]. For example, at a hospital in Brazil, de Pina Carvalho et al. [101] displayed that the charge of successful treatment with the first-line drugs (pentavalent antimony) for each patient was calculated at US$ 167.7, for miltefosine was US$ 259.9, and for AmBisome® (liposomal amphotericin B), it was US$ 715.4. In many assessments, the cost of drugs has been reported as more than 60% of the overall cost for each treatment process [101]. The budget provision of medicines in a sustainable way is a difficult challenge for health policymakers in many countries [102]. The health budget is often a substantial fundamental section of national budgets. It is generally one of the significant parts of the general government budget. In 2016, in the 28 countries belonging to the EU since 2007, the total cost of health was 7% of the gross domestic product [103]. Healthcare expenditure continues to develop at rates that overstep the government's income growth, increasing problems regarding the future financial sustainability of the healthcare system [103]. It is worth noting that in general in most countries, leishmaniasis is considered a neglected disease that has lower importance than other important diseases, and consequently, a lower budget belongs to it.

Surveys in Iran have shown that the initial health requirements are prepared by the health clinics and completed by the expert staff and physicians. Tertiary hospitals are in charge of preparing secondary healthcare facilities for residents. The cost of medical services was raised in the last decade, however, there is still a lack of budget for healthcare [104, 105].

The number of current health centers and expert staff is not sufficient in some endemic regions, health staff often have a low experience, provisions are not enough, and also not enough medicines. Lack of adequate budget and intersectoral cooperation, absence of local and international consideration to control plans, and associated subjects are often worrying. As regards the burden of disease has varied magnitudes, and this needs great efforts and measures from the government including policymakers and healthcare management, and local and international organizations [11].

#### 3.2.2. Proper and Prompt Diagnosis

In general, diagnosis of clinical leishmaniasis relies on the clinical ground, epidemiological information, and parasite identification by direct microscopic smear preparation of skin tissue samples and culturing the organism for CL. Parasitological confirmation at the species level using the more sensitive intrinsic tests such as molecular, immunological, or biochemical assays is not a routine practice; although might be practical in some countries [106].

#### 3.2.3. Follow-Up Assessments

The follow-up examination of treated leishmaniasis cases is crucial to determine the effectiveness of treatment [107]. Also, the healthcare systems may reach more success by increasing the number of clinical visits and follow-ups, these positive efforts can be associated with progress in adherence to treatment [108]. In ACL endemic areas, usually, there is no appropriate follow-up effort for cases with CL following the treatment course. Some cases are absent from the treatment follow-up assessment which further contribute to the recurrence of cases [109].

In other diseases for example diabetes mellitus, the findings of a study displayed that personal support assembly education and 12 weeks of follow-up examination via a health worker using the telephone led to considerable progress in the metabolic indicators and treatment adherence [110]. The deficiency of accordance between patient preparation and the physician's efforts at intervention means that treatments are often prescribed to cases that are not ready to follow their treatment. The primary challenges that can cause poor follow-up are the nonaccessibility of drugs, great distance to health clinics, incomplete and irregular treatment, and job limitations. Healthcare providers should be able to determine the patient's preparation to adhere, provide consultation on how to perform it, and follow-up on the patient's improvement every contact [47]. Such follow-up examinations help with proper treatment and reduce unresponsive cases.

#### 3.2.4. Health Personnel Training and Public Education

Training courses and improving skills for healthcare personnel on several aspects of the diseases, including early diagnosis, management of cases, various control strategies, and prompt response to outbreaks should be done constantly for control of CL. Confident education for healthcare staff is a very cost-effective preventive procedure [111–113]. In a study, health education provided the community with important visions on disease transmission (life cycle of parasites in vectors, reservoirs, and humans), which resulted in environmental management approaches. These changes in approaches led to a noticeable decrease in the vector population in their region [98]. In endemic areas, informing the public lead to proper case detection, and better admission of therapeutic and preventive proceedings.

On the other hand, education on the proper use of insecticide–impregnated dresses and curtains, and diagnostics, training procedures of CL for healthcare staff are necessary [114, 115]. Society and patients should be educated and knowledgeable about the disease, control and prevention actions, and access to proper healthcare services. Common practices for the diagnosis and management of leishmaniasis should be expanded by circulating strong communications between healthcare staff and patients. The healthcare system should be used for education that precisely aims at society's rise in community participation and effect to enhance control measures of leishmaniasis in marginalized parts of endemic areas [3].

Also, in some countries with diverse cultures, language barriers are a challenging obstacle for the healthcare system in training and public education [116–118]. In other infectious diseases, trials to evaluate the effect of intensive health education and counseling on patients with active tuberculosis showed increasing treatment completion rates for the cases who took intensive education and counseling compared with cases who received routine care [119]. Education of endemic populations in the etiology of the disease, transmission, treatment, and control measures has a vital role to play in the managing, prevention, and control strategies of leishmaniasis. Additional comprehensive studies are required to access the influence of community education in the control and prevention measurements.

#### 3.2.5. Optimal Reporting

Many inadequacies affect the control strategies. There are still major problems that possibly play a confusing role in the ideal delivery of medical services. The main formal facts are passive case finding that is presented through the public health surveillance (PHC) network. Some cases of the disease are not reported, misdiagnosed, or undiagnosed, particularly in poor villages and remote widespread regions. Most of the cases due to various reasons (work constraints) are not reported. Several individuals are subclinically infected (asymptomatic and remain untreated in the area). They may induce the transmission of causative *Leishmania* species. The proportion of subclinical infections (asymptomatic) to clinical forms is believed to vary and it is fairly high in the endemic localities. A lack of knowledge related to these factors can further complicate the conditions and intensification of the disease [3, 106].

#### 3.2.6. Early Detection and Effective Treatment

Clinical examination of CL lesions, early detection of infection, and identification of causative *Leishmania* species are vital for choosing an appropriate therapy and designing proper control strategy, particularly in ACL caused by *L. tropica* in the endemic foci [16, 109]. In these regions, control strategies are restricted to initial diagnosis and immediate therapy of cases. In a study, CL cases who began their treatment after 12 months due to late detection or other related reasons, had a higher potential for the increase of treatment failure forms than those who had timely treatment [16].

The morphology of CL lesions takes mistakes with a wide range of viral diseases such as herpes-like, zoster, fungal diseases, and wart viruses, sporotrichosis and lupus vulgaris, bacterial diseases such as mycobacterial ulcers and tuberculosis, and skin diseases such as myiasis, tropical ulcers, foreign-body granuloma, ecthyma, sarcoidosis as well as the number of parasitic diseases and malignancies. Awareness of such similar morphological characterization and the exact diagnosis according to demonstration and observation of the parasite is necessary for any treatment and care in the endemic regions [3, 24, 31, 40, 109].

ZCL (wet type) and ACL (dry type), make up various forms of CL with diverse appearances worldwide. Some unusual clinical forms of CL are connected with various parasite species and the host's immunity. The usual stages of progress for the cutaneous lesion are papule, nodule, ulcer, and scar [120].

Management of ACL due to *L. tropica* mainly depends on prompt diagnosis, identification of the species, and proper and on-time treatment by a surveillance system [109]. Considering that a human is an alone reservoir host, unresponsive chronic patients such as recurrence cases (relapse) remain the reservoir of infection for another individual [121, 122]. So, surveillance personnel have a vital duty in this important issue for the public.

As already mentioned, leishmaniasis control depends on the several approaches. The quantity and quality of the drug are also other determinants that can significantly affect the treatment efficiency.

Poor adherence to the proper treatment happens to different causes, such as uncertainty about the predictable effectiveness, the low efficacy of therapy, unsuitable adverse effects (musculoskeletal pain, gastrointestinal disturbances, headache, mild dyspnea, erythema, and allergic reactions), work restriction or socioeconomic status, traveling or relocation from their living place, depression, and forgetfulness [47, 123].

Resistance or treatment failure in CL is a complicated and multifactorial occurrence. Hence, necessary, and comprehensive actions by the healthcare system and policymakers are vital to ensure that patients follow health orders well. Prompt detection and proper treatment after the beginning of the lesion are important for the effective treatment of the cases [40, 64].

#### 3.2.7. Handling of Unresponsive Cases

Leishmaniasis is a curable disease and is treated with pentavalent antimonial drugs, but failure and unresponsiveness to these medicines have remarkably raised in the endemic foci worldwide [31, 124]. Treatment failure is an important challenge reported for many years in treating and controlling CL. Several determinants have assisted this challenge, including the demographical, clinical, and environmental factors, poor adherence to treatment, the immune system of the host, the genetic composition of the *Leishmania* species, and the anthroponotic (human-to-human) transmission [109, 125, 126].

ACL due to *L. tropica* being limited to individuals as an anthroponotic form and drug-resistant is a considerable task for the appropriate treatment of CL cases. This challenge requires effective treatment modalities and the improvement of new drugs or combination therapy for CL. Based on the previous studies, various treatment modalities have been used for CL [127–130]. The findings of a study done in Iran demonstrated that a combination of levamisole plus MA is effective and could be considered in unresponsive cases with ACL who previously have not responded to standard treatment (MA) [131]. Since human is the sole reservoir of parasite and disease, therefore, the cases should be treated regularly to avoid further distribution of the parasite by sandflies to other people. However, in the ZCL type, cases with treatment failure are reported in endemic foci, and in this condition, humans can be incidental reservoir hosts, but probably these cases have no role in the transmission of the disease [40, 126]. The main preventive actions for creating the treatment failure forms and relapse are including more follow-up examinations, further education, and preventive procedures to protect high-risk individuals and decrease their exposure to human vectors. These unresponsive forms (including parasite resistance and immune deficient patients) also underscore an essential need for novel effective medicines against ACL for the planning of future therapeutic and control strategies [109]. Overall, the healthcare system should identify and handle treatment failure cases and carry out the necessary interventions including complete adherence to treatment, use of combined treatment approaches, improving patients' awareness about the unresponsive forms, and giving hope to patients with stress and anxiety induced by the disease for effective treatment.

#### 3.2.8. Active Case Detection

Active case finding through house-to-house visits is an essential part of the leishmaniasis elimination strategy that systematically screens the population to find cases. It would help reduce disease spread by reducing the infectious period of cases. In a study, active case detection in VL cases significantly reduced the time from the onset of fever to diagnosis [132]. Studies revealed that the cost of early detection of further VL and CL cases is lower than outweighs the long-term costs for achieving VL and CL elimination benchmarks [133, 134]. Also, active case detection is cost-effective in regions where the incidence rate is high [133]. Furthermore, active case detection and early treatment of ACL cases could be effective approaches to controlling the disease, especially in times of epidemics such as in the earthquake in Bam, Iran [135]. Generally, in endemic areas, the awareness of the population regarding the disease is inadequate and health services have weak activities. There are some active case detection tactics such as house-to-house search (or blanket screening), camp approach, index case approach (or “snowballing”), and an incentive-based approach [136]. Active case detection is a crucial measure in decreasing the incidence rate of CL under outbreak situations. Although most healthcare systems depend on passive case detection, CL active models should be investigated and used. Screening of CL by health workers is done based on the recognized risk factors. These factors include the history of travel to endemic areas, duration of skin lesions, gender, and profession, and finally, the cases are guided to centers where the diagnosis is accurate, which may help better CL control [109]. To the lack of active case findings, low knowledge among the people, and an inappropriate health care system, the disease is detected late and consequently creates problems for achieving the proper controlling programs.

#### 3.2.9. Monitoring and Evaluation

Monitoring is the general check-out investigation and consideration of the plan for the control and prevention of CL. These examinations include collection and recording of the data, analysis, reporting, improvement of practical preparation, and assessment of community health functions. The foremost aim of monitoring is to measure the development and operation situations, find essential challenges and restrictions, and finally decision-making according to the present report [61, 137]. The monitoring and management of the CL are according to the directory of predetermined indicators (epidemiological and operational) used. These indicators are usually applied in evaluating the program's performance. These will be effective and practical with empirical information on diverse stages of the control strategy at different local and international levels [7, 61, 137].

## 4. Discussion

**Figure 3:**
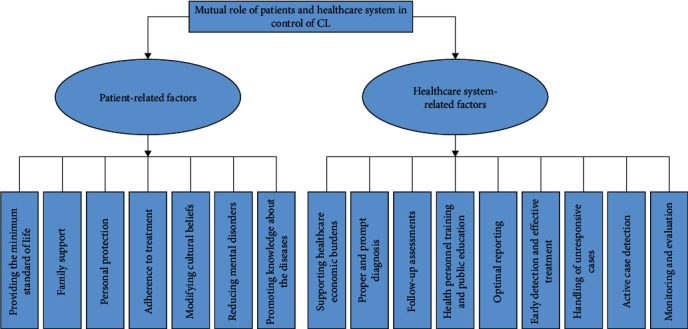
Two significant determinants for the control of CL include patient-related factors and healthcare system-related factors.

Over the years, CL has noticeably increased and various epidemics happened due to many complex determinants [15, 126, 138–142]. Leishmaniasis has no effective drugs nor an efficacious and affordable vaccine. On the other hand, control of sandfly vectors and reservoirs is not eco-friendly and practical. Therefore, the importance of controlling the disease by recognizing all aspects; gaps, and challenges, especially two significant determinants including patients and the healthcare system, and their suitable interaction with each other is highly essential. Each of these two merits entails several other irrelevant factors that are in brief mentioned in the text and also the challenges that arise from them were explained. The interesting thing to note is that all these factors are closely related to each other and each of them alone and along with other aspects can play a significant role in controlling the disease. Some patient-related factors that have a remarkable effect on proper treatment and control are poverty, poor socioeconomic situation, low level of education, illiteracy, unstable conditions of living, unemployment, lack of sufficient knowledge about the disease, a long distance from the treatment center, high cost of drugs, high cost of transportation, culture and incorrect beliefs about disease and therapy, depression and lack of proper support from the family (Figure 3) [47].

**Figure 4:**
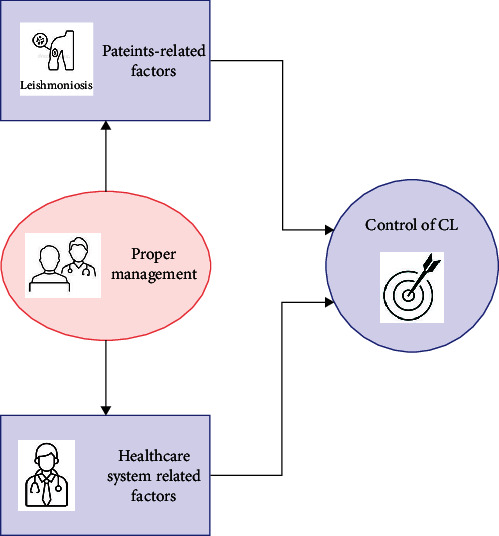
Proper management between the two main determinants for control of cutaneous leishmaniasis (the patient and the healthcare system).

On the other hand, some healthcare-associated factors that have an important impact on proper therapy and control strategy are the economic burden of healthcare and drug preparation, unsuitable follow-up assessments, health education, early detection, and proper treatment, handling of unresponsive cases, active case detection, and monitoring (Figure 3). The correct management of the two main determinants of the disease (the patient and the health-surveillance system) can lead to the control of the disease (Figure 4).

The policymakers have extremely ignored CL, and health networks do not consider CL a serious risk and crucial sanitation problem worldwide. While leishmaniasis is the ninth major disease burden in terms of morbidity and mortality among infectious diseases, it is mainly overlooked because of its multifaceted epidemiology and ecology, inadequate practical tools regarding its case management, and the insufficiency of present surveillance systems.

To our knowledge, this review is unique, and no similar study has previously been published to entirely highlight related mutual aspects of patients and the healthcare system in the precise management of the disease. Therefore, despite comprehensive searches, the main limitation of this study was the lack of enough sources on different aspects of CL control in the endemic areas.

It is necessary to mention that due to the complexity of the disease, major challenges and gaps remain till further innovative tools become available before CL can ultimately be controlled.

## 5. Conclusion

In a suitable strategy, it is essential to precisely understand all aspects of control plans, particularly the mutual role of patients and the healthcare system to respond correctly to the requests in the different endemic regions. Trained personnel and experienced clinicians should strengthen capacity building for appropriate control strategies throughout the health and medical units. Improving socioeconomic essentials and quality of life will support prolonged control approaches and facilitate the exclusion of the disease. This will need consistent encouragement and appropriate activities through all governmental sectors such as policymakers, the health system, clinical physicians, researchers, and private parties. Also, the advancement of public awareness about the disease, early case detection, proper treatment, and providing sufficient diagnostics and therapeutics on a long-term basis can help improve the control strategies of the disease. Since the literature on the related topic is rudimentary, further intimate experiences are highly recommended to manage CL patients properly.

## Data Availability

All data generated through the review process is presented within the manuscript.
